# Effect of an Intensified Combined Electromyography and Visual Feedback Training on Facial Grading in Patients With Post-paralytic Facial Synkinesis

**DOI:** 10.3389/fresc.2021.746188

**Published:** 2021-10-14

**Authors:** Gerd F. Volk, Benjamin Roediger, Katharina Geißler, Anna-Maria Kuttenreich, Carsten M. Klingner, Christian Dobel, Orlando Guntinas-Lichius

**Affiliations:** ^1^Department of Otorhinolaryngology, Jena University Hospital, Jena, Germany; ^2^Facial Nerve Center, Jena University Hospital, Jena, Germany; ^3^Department of Neurology, Jena University Hospital, Jena, Germany

**Keywords:** facial nerve paralysis, synkinesis, biofeedback, electromyography, rehabilitation, physical therapy

## Abstract

**Background:** There is no current standard for facial synkinesis rehabilitation programs. The benefit and stability of effect of an intensified 10-day facial training combining electromyography and visual biofeedback training was evaluated.

**Methods:** Fifty-four patients (77.8% female; median age: 49.5 years) with post-paralytic facial synkinesis (median time to onset of paralysis: 31.1 months) were included in retrospective longitudinal study between January 2013 and June 2016. Facial function was assesses at baseline (T0), first days of training (T1), last day of training (T2), and follow-up visit (T3) at a median time of 6 months later using the House-Brackmann (HB) facial nerve grading system, Stennert index (SI), Facial Nerve Grading System 2.0 (FNGS 2.0), and Sunnybrook Facial Grading System (SFGS). Pairwise comparisons between the time points with *post-hoc* Bonferroni correction were performed.

**Results:** No significant changes of the gradings and subscores were seen between T0 and T1 (all *p* > 0.01). The 10-day combined and intensified feedback training between T1 and T2 improved facial symmetry and decreased synkinetic activity. Facial grading with the FNGS 2.0 or the SFGS were most suited to depict the training effect. FNGS 2.0, regional score, FNGS 2.0, synkinesis score, and FNGS 2.0 total score improved significantly (all *p* ≤ 0.0001). Both, the FNGS 2.0 and the SFGS showed the strongest improvement in the nasolabial fold/zygomatic and the oral region. Neither the age of the patient (*r* = 0.168; *p* = 0.224), the gender (*r* = 0.126; *p* = 0.363) nor the length of the interval between onset of the palsy and training start (*r* = 0.011; *p* = 0.886) correlated with the changes of the SFGS between T1 and T2. The results remained stable between T2 and T3 without any further significant change.

**Conclusion:** Intensified daily combined electromyography and visual biofeedback training over 10 days was effective in patients with facial synkinesis and benefits were stable 6 months after therapy.

## Introduction

Post-paralytic facial synkinesis is a disfiguring condition characterized by involuntary contraction of one or more facial muscles during voluntary movement of other facial muscles (1). About 30–40% of patients with acute facial palsy do not recover completely and develop synkinesis (2). Problems with eye closure and eating, the inability to smile and affected face-to-face communication are the major motor impairments and non-motor symptoms leading to decreased quality of life (3). Nevertheless, many patients with synkinesis are never referred to a specialist or with significant delay (4). Besides botulinum toxin treatment, physical rehabilitation therapy is the most often prescribed measure (1, 5). The efficacy of physical therapy is very heterogeneous within the same study and between studies (6), because physical therapy types, schedules, frequency, and duration are highly variable and not standardized (5). Furthermore, facial palsy can be caused by a variety of diseases influencing the outcome and also the effect of physical therapy (7, 8).

The primary aim of a neuromuscular facial biofeedback training is that the patient learns how to change facial muscle activity of the affected side for the purpose of improved facial function (9). Facial biofeedback training mainly uses surface electromyography (EMG) recording of facial muscle activity and a feedback by visualization or acoustic signals (10–14). Astonishingly, the daily training periods in the literature take only 30–60 min distributed to a few sessions per week. Pathological recovery after deefferentation without deafferentation in case of facial paralysis is a complex disorder (15). From constraint-induced movement rehabilitation programs for patients after stroke, it is well-known that a daily forced use of an affected extremity for several hours per day over 2–3 weeks is needed to overcome corticomotor suppression and mismatch (16–18).

Therefore, we established in 2012 an intensified combined electromyography and visual feedback training program for patients with post-paralytic facial synkinesis after various etiologies of facial palsy (9). A pilot study with 20 patients using instructed raters revealed significant improvements of facial movements after the training (19). Here, we wanted (1) to confirm these encouraging results based on validated facial grading systems and additionally to test the hypotheses that (2) facial grading does not improve during the waiting time to facial training, and that (3) facial grading shows stable therapy effects over 6 months after facial training.

## Methods

### Study Design and Inclusion Criteria

This retrospective observational and longitudinal study included patients with post-paralytic facial synkinesis after various etiologies of facial palsy who presented in the Facial Nerve Center, Jena University Hospital, between January 2013 and June 2016. All facial palsy related data and questionnaires were collected in the Facial Nerve Center. The inclusion criteria were: (a) a unilateral peripheral facial palsy; (b) an interval between onset and assessment of at least 6 months; (c) facial electromyography (EMG) confirmed voluntary activity in the affected facial muscles including synkinetic activity (20).

### Intensified Combined Electromyography and Visual Biofeedback Training of Facial Movements

**Figure 1:**
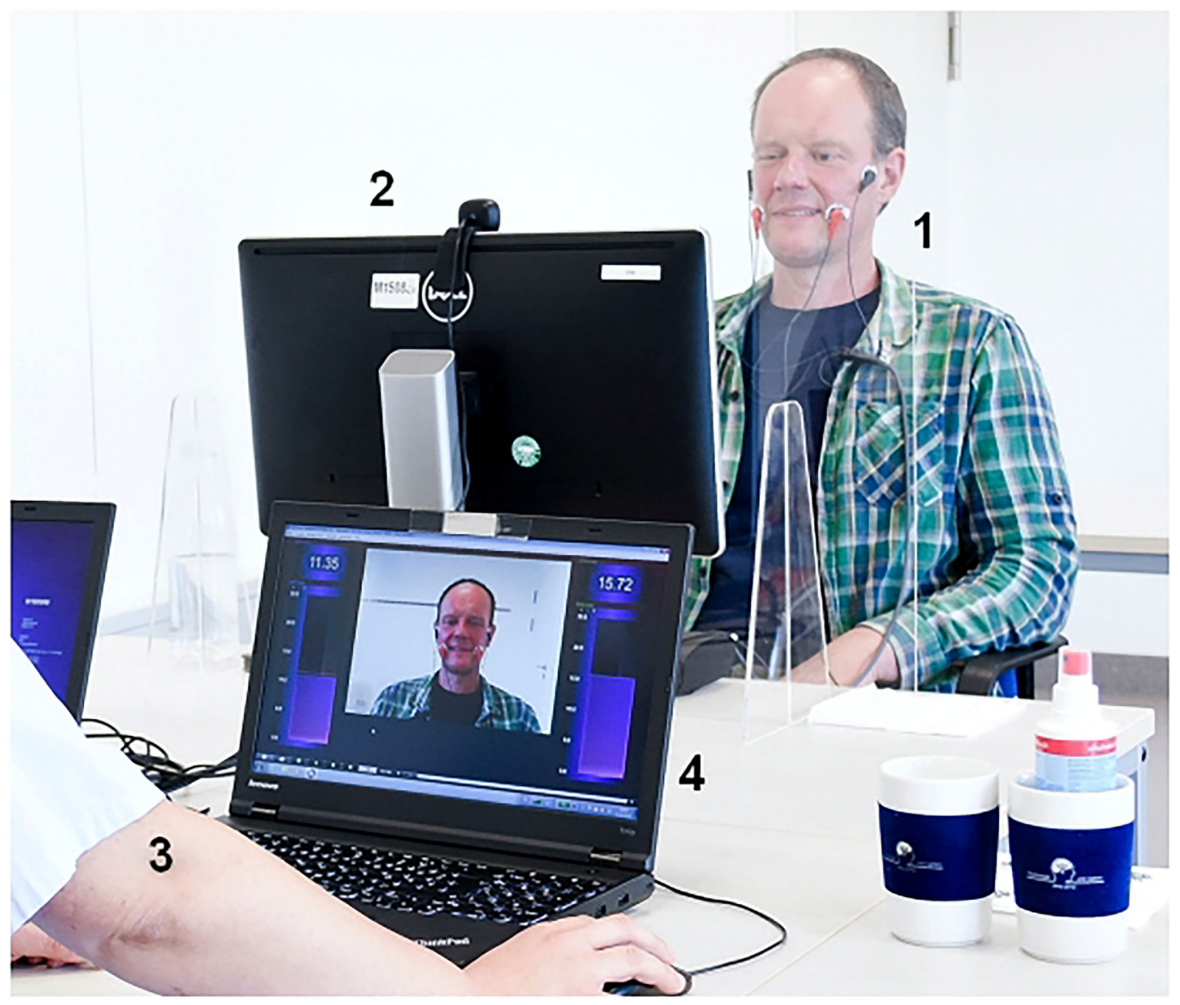
Combined visual and EMG biofeedback setting. The patient (1) is sitting in front of a screen (2) showing himself via a camera on top of the screen. The therapist (3) is sitting in opposite to the patient allowing a direction view on patient's face. Beyond the face of the patient, the screen of the therapist (4) is showing the same information as the patient's screen: Bars are showing the surface EMG activity of the recorded muscles, in this examples the EMG activity is recorded symmetrically from both zygomatic muscles to control lifting the corner of the mouth on the affected side symmetrically to the contralateral side.

The training was carried out over a period of 10 days (two times for 5 days, the weekend in between without therapy). In the mornings, under the guidance of a therapist, 3 h of intensive facial training with (EMG) biofeedback combined with elements of constraint induced movement therapy was performed (9, 17–19). Biofeedback training was performed using the Nexus 10 biofeedback system, with Bio Trace software animations (Mind Media BV, Netherlands). Briefly, the patient was trained to control a defined and isolated facial muscle movement (for instance, pursing the lips by activation of the orbicularis oris muscle) without moving other facial muscles (for instance, without synkinetic activity of the ipsilateral orbicularis oculi muscle). To give another example, a specific activation of the zygomatic muscles on one or on both sides was performed while avoiding or at least minimizing synkinetic activation of the ipsilateral orbicularis oculi muscle. Surface EMG was recorded simultaneously and bilaterally from the target muscle for the intended movement and the most important muscle of an unintended movement (Figure 1). More details are given in Supplementary Table 1. Together with a video-generated mirror image, the patient could then simultaneously track the muscle activity on a screen during her/his movement exercises. The muscle activity was visualized with EMG feedback bars. The feedback signal was always proportional to the muscle activity. Due to the EMG feedback, the patient could track very fast voluntary and involuntary facial muscle activities even in their smallest forms. In this way, even unconscious movements were shown to the patient. A conscious relaxation of the muscles before and between every movement exercise was promoted on that way. The therapist was sitting opposite to the patient. So, the therapist could directly observe the patient and at the same time sees on the computer screen the video picture of the patient and the EMG feedback bars in the same way as the patient itself sees it on her/his screen. Thereby, the therapist could see the feedback, progress and deficits easily and could fast adapt the movement exercises if needed. The aim was that the patients developed new movement patterns in order to reduce synkinesis, control muscles independently and in this way balance their activity. Each afternoon, the patients performed an independent training for 2 h using a hand mirror. The patient documented the afternoon training on an exercise sheet (Supplementary Material). This was based on tasks and exercises first trained with the therapist. These exercises were inspired by a facial training booklet visualizing a standard set of facial exercises (21). The patients were encouraged to continue the exercises they had learned at home for at least 30 min daily for the following 6 months. No facial-palsy specific training was allowed during the waiting time before the training. Furthermore, no facial surgery or botulinum toxin injections were allowed between T0 and T3.

### Measurement Times and Facial Grading

**Figure 2:**
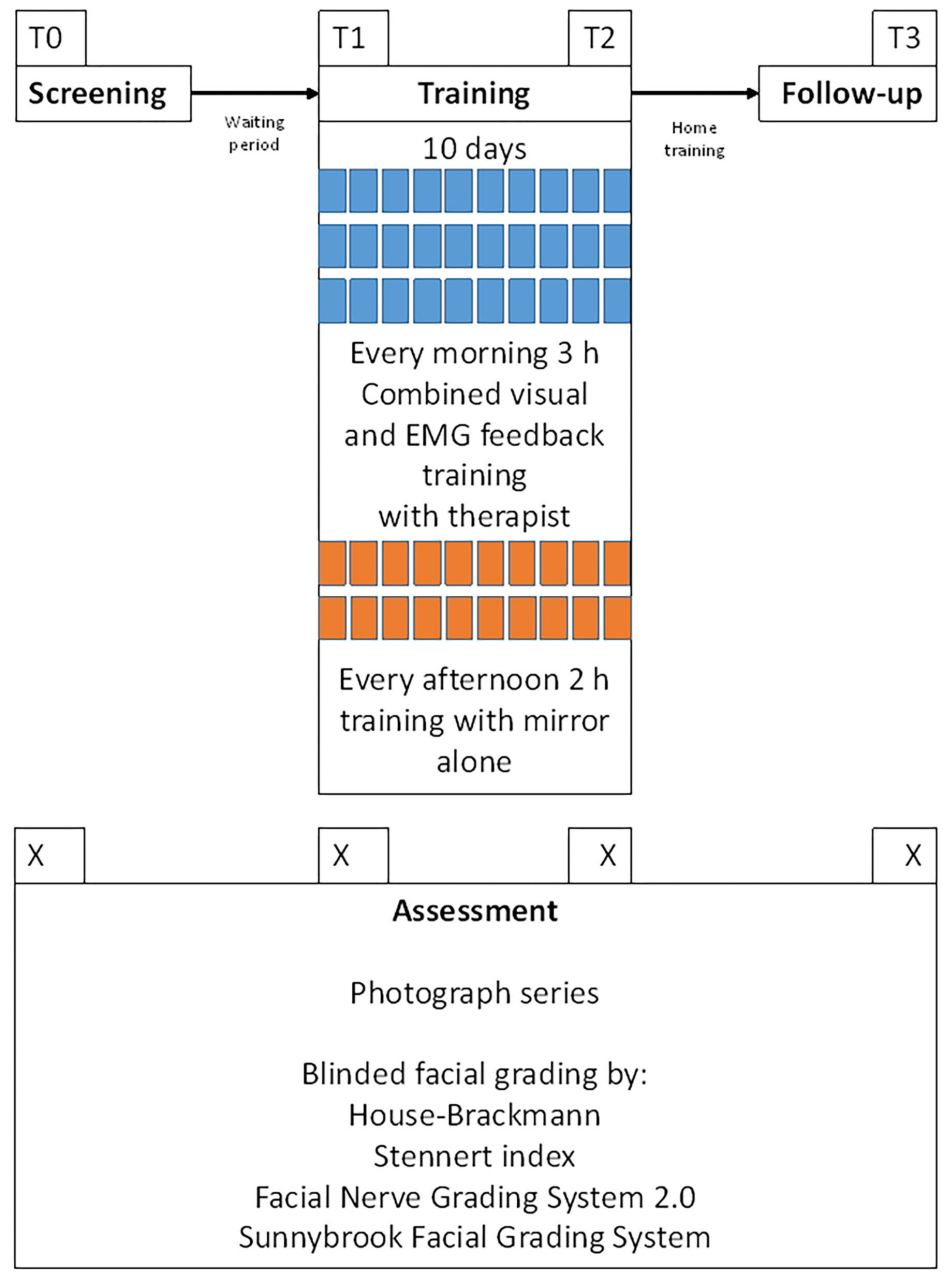
Workflow of the examinations. T0 = day of the screening and inclusion into the training and the study. T1 = first day of the facial training. T2 = last day of the facial training (10th day). T3 = follow-up examination. Median interval between T0 and T1 (waiting period) was 4.4 months. Median interval between T2 and T3 (home training period) was 6 months.

Patients' charts were reviewed for demographic characteristics, patients' history, and prior treatment. All grading assessments were performed at four points in time: T0 = screening day and inclusion; T1 = start of facial feedback training; T2 = last day of the training, and T3 = follow-up examination at a minimum of 6 months later. Figure 2 shows the examination workflow. Uniform series of photos were taken for all 54 patients for an objective assessment of facial function (22). Briefly, a sequence of static posed nine expressions was always photographed: (1) at rest, (2) closing both eyes, (3) closing both eyes with maximal effort, (4) frowning, (5) wrinkling the nose, (6) lifting both corners of mouth with closed mouth, (7) showing the teeth, (8) pursing the lips, and (9) pull down both corners of mouths. Hence, nine images were taken as a set per patient per time of assessment. Before evaluation, all photographs were blinded for the measurement time. The rater (BR) was not involved in the recruiting nor the training of any of the patients. He is a medical doctor in the training to a maxilla-facial surgeon with several years of experience in grading facial paly patients.

As shown in Supplementary Table 2, four different facial grading systems were used to classify facial nerve motor function based on the photographs described above. Grading was performed by the House-Brackmann (HB) facial nerve grading system, Stennert index (SI), Facial Nerve Grading System 2.0 (FNGS 2.0), and Sunnybrook Facial Grading System (SFGS). The HB ranges from grade I (normal function) to grade VI (complete paralysis) (23). In contrast, the SI is a double-weighted system (24). The observer judges facial symmetry at rest in four categories (0 =normal resting tone/symmetry up to 4 = no resting tone/gross asymmetry) and the motility of the facial muscles in six categories (0 =normal motility up to 6 = complete paralysis). The total score of the Stennert index summarizes both subscores. The FNGS 2.0 is a further development of the House-Brackmann facial nerve grading system (25). The FNGS 2.0 determines the final grade by adding regional assessments (score from 1 to 6) of the brow, eye, nasolabial fold, and oral regions to the score assessing the impact of synkinesis (score from 0 to 3). Summation of scores gives a final score of 4–24. Finally, the SFGS is a regional weighted system that rates three subscores (26): resting symmetry, the degree of voluntary facial muscle movement, involuntary muscle contraction (synkinesis). The three subscores are used to calculate a composite score (0 = total paralysis; 100 = normal function).

### Statistical Analysis

The statistical analysis was performed with SPSS version 25.0 (IBM, Armonk, NY, USA). If not indicated otherwise, data are presented with mean values and 95% confidence intervals (CI). The comparisons between T0 and T1 (changes in the waiting period without any facial palsy specific intervention), between T1 and T2 (therapy effects), and T2 and T3 (changes during follow-up with active daily self-training) were performed with the non-parametric Wilcoxon test for paired data. These three, i.e., multiple comparisons were corrected with the Holm-Bonferroni method. Therefore, the corrected significance level was set at *p* < 0.0001. Cohen's d for paired data was calculated to evaluate the effect size between means of two measurement points. A large effect size was defined as *d* ≥ 0.8. The Spearman test was used bivariate correlation analyses between different facial grading systems as well as to analyze the correlation of the changes of facial grading between T1 and T2 vs. age, gender, or duration of the palsy. The significance level was set at *p* < 0.0001.

## Results

### Patients' Characteristics

A total of 54 patients were included (77.8% female; median age: 49.5 years). More details are shown in Table 1. The median interval between onset of the facial paralysis and training start was 31.1 months. The median interval between screening (T0) and start of the training (T1) was 4.4 months. Median interval between end of the training (T2) and follow-up examination was 6.0 months.

**Table 1:** Patients' characteristics.

**Parameter**	**Absolute (*N*)**	**Relative (%)**
All	54	100
**Gender**		
Female	42	77.8
Male	12	22.2
**Localization**		
Left	30	55.6
Right	24	44.4
**Etiology**		
Idiopathic	23	42.6
traumatic/post-surgical	18	33.3
Inflammatory	12	22.2
Stroke, brainstem	1	1.9
	**Mean** **±** **SD**	**Median, range**
Age, years,	42.8 ± 1.5	49.5, 14–77
Interval onset of facial palsy to training, months	62.3 ± 66.9	31.1, 12–302
Interval T0–T1 (waiting period), months	4.8 ± 2.4	4.4, 0.7–10.5
Interval, T2–T3 (follow-up period), months	6.3 ± 1.3	6.0 ± 5.0–11.8

### Facial Nerve Grading in the Time Course From T0 to T3

**Figure 3:**
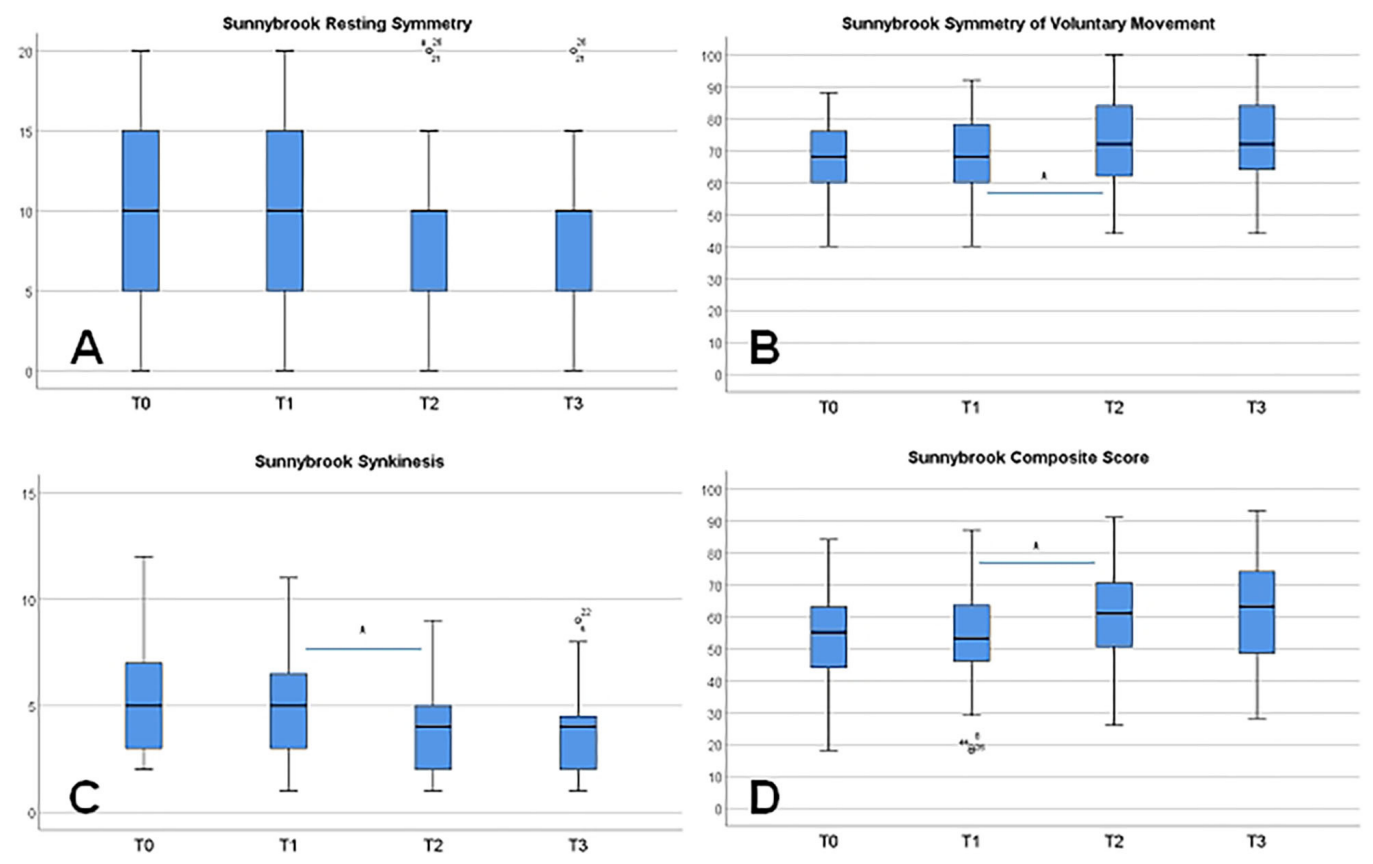
Changes of the facial grading from T0 to T3 using the Sunnybrook grading scale and its subscores. **(A)** Symmetry of the face at rest. **(B)** Symmetry of voluntary movements. **(C)** Synkinesis. **(D)** Composite score. Significant changes (*p ≤ 0.001) are only seen between T1 and T2 as a training effect, except for symmetry at rest.

All four grading systems confirmed the notable chronic facial movement disorder of the patients at baseline (Table 2) Grading with the FNGS 2.0 and the SFGS confirmed the detection of relevant synkinesis. No significant changes of the gradings and subscores were seen between T0 and T1. On average, the 10-day combined and intensified feedback training between T1 and T2 improved facial symmetry and decreased synkinetic activity. This was statistically most significantly obvious when using the FNGS 2.0 or the SFGS for facial grading (Figure 3). FNGS 2.0, regional score, FNGS 2.0, synkinesis score, and FNGS 2.0 total score improved significantly (all *p* ≤ 0.0001). The median improvement between T1 and T2 using the SFGS was 7 points (range: −4 to 21). Strong effect sizes could be calculated when analyzing the changes measured by the SFGS (*d* = 1.36) and the FNGS 2.0 (*d* = 1.15) (Table 2).

**Table 2:** Comparison of facial grading at the initial screening (T1), start (T1), and end (T2) of therapy, and at follow-up (T3)^*^.

**Parameter**	**T0**		**T1**		**T2**		**T3**		**T0–T1**	**T1–T2**	**T2–T3**
	**Mean**	**CI**	**Mean**	**CI**	**Mean**	**CI**	**Mean**	**CI**	**p; d**	**p; d**	**p; d**
House-Brackmann	3.1	2.9–3.3	3.1	2.9–3.3	2.8	2.8–3.0	2.9	2.7–3.1	0.046; 0.29	**≤0.001**; 0.68	0.156; 0.19
Stennert, rest	1.4	1.2–1.6	1.4	1.1–1.6	1.2	1.0–1.4	1.2	1.0–1.4	0.532; 0.09	0.021; 0.46	0.766; 0.04
Stennert, motion	2.6	2.2–2.9	2.6	2.2–2.9	2.4	2.1–2.9	2.4	2.1–2.4	0.766; 0.77	**≤0.001**; 0.72	0.485; 0.10
Stennert, total	3.9	3.5–4.4	3.9	3.5–4.4	3.6	3.1–4.1	3.6	3.2–4.1	0.857; 0.04	0.007; 0.54	0.821; 0.03
FNGS 2.0, brow	4.4	3.9–4.9	4.4	4.0–4.9	4.3	3.8–4.7	4.4	3.9–4.8	0.010; 0.38	0.002; 0.64	0.058; 0.26
FNGS 2.0, eye	1.7	1.4–1.9	1.6	1.4–1.9	1.6	1.3–1.8	1.5	1.3–1.8	0.444; 0.11	0.160; 0.28	0.569; 0.08
FNGS 2.0, NLF	3.1	2.7–3.4	2.9	2.6–3.2	2.5	2.2–2.8	2.4	2.1–2.7	0.038; 0.30	**≤0.001**; **0.84**	0.419; 0.11
FNGS 2.0, oral	2.2	2.0–2.4	2.2	2.0–2.4	1.9	1.7–2.1	1.9	1.7–2.1	0.766; 0.04	**≤0.001**; 0.70	0.709; 0.05
FNGS 2.0, regional	11.3	10.4–12.2	11.2	10.3–12.0	10.2	9.4–11.0	10.2	9.4–11.0	0.597; 0.08	**≤0.001**; **1.13**	0.830; 0.03
FNGS 2.0, synkinesis	1.5	1.4–1.7	1.5	1.3–1.7	1.2	1.1–1.3	1.3	1.1–1.4	0.659; 0.06	**≤0.001**; 0.70	0.419; 0.11
FNGS 2.0, total	12.8	11.9–13.7	12.7	11.8–13.5	11.4	10.6–12.2	11.4	10.7–12.2	0.909; 0.02	**≤0.001**; **1.15**	0.569; 0.08
SFGS, resting symmetry	9.4	7.9–10.9	9.6	8.2–11.1	8.5	7.2–9.9	8.4	7.0–9.8	0.799; 0.04	0.003; 0.60	0.821; 0.03
SFGS, frontalis	2.3	1.9–2.6	2.2	1.9–2.5	2.4	2.0–2.7	2.3	2.0–2.6	0.010; 0.38	0,005; 0.57	0.322; 0.14
SFGS, orbicularis oculi	4.4	4.2–4.7	4.4	4.2–4.7	4.5	4.3–4.7	4.5	4.3–4.7	07.85; 0.03	0.261; 0.22	0.569; 0.08
SFGS, zygomaticus, risorius	3.1	2.8–3.4	3.2	3.0–3.5	3.6	3.3–3.9	3.7	3.4–4.0	0.006; 0.40	**≤0.001**; **0.81**	0.013; 0.35
SFGS, levator labii superior	3.1	2.8–3.4	3.2	3.0–3.5	3.6	3.3–3.9	3.6	3.3–3.9	0.051; 0.28	**≤0.001**; **0.91**	1.000; 0.00
SFGS, orbicularis oris	3.9	3.7–4.1	3.9	3.7–4.1	4.4	4.0–4.4	4.2	4.1–4.4	0.532; 0.09	**≤0.001**; 0.72	0.419; 0.11
SFGS, movement symmetry	67.0	63.1–70.9	68.0	64.4–71.6	73.0	69.3–76.6	73.5	69.8–77.1	0.243; 0.16	**≤0.001**; **1.15**	0.212; 0.17
SFGS, synkinesis frontalis	0.9	0.7–1.1	0.9	0.7–1.1	0.7	0.6–0.9	0.7	0.6–0.9	0.766; 0.04	0.021; 0.46	0.742; 0.05
SFGS, synkinesis orbicularis oculi	0.6	0.4–0.7	0.6	0.5–0.7	0.4	0.3–0.6	0.5	0.4–0.7	0.742; 0.05	0.003; 0.60	0.013; 0.35
SFGS, synkinesis zygomaticus, risorius	1.3	1.2–1.5	1.3	1.1–1.5	1.1	1.0–1.3	1.1	0.9–1.3	0.420; 0.12	0.021; 0.46	0.410; 0.11
SFGS, synkinesis levator labii superior	1.3	1.1–1.5	1.3	1.1–1.5	1.0	0.9–1.3	1.0	0.8–1.2	0.322; 0.14	**≤0.001**; 0.68	0.532; 0.09
SFGS, synkinesis orbicularis oris	1.0	0.7–1.2	0.9	0.7–1.2	0.5	0.3–0.7	0.5	0.4–0.7	0.532; 0.09	**≤0.001**; **0.89**	0.485; 0.10
SFGS, synkinesis	5.1	4.4–5.8	5.0	4.4–5.6	3.8	3.3–4.4	3.9	3.3–4.4	0.424; 0.11	**≤0.001**; **1.04**	0.808; 0.03
SFGS, composite	52.5	48.1–56.9	53.4	49.2–56.6	60.6	56.5–64.7	61.2	57.0–65.4	0.520; 0.13	**≤0.001**; **1.36**	0.322; 0.13

* *Significant/strong effects in bold; FNGS 2.0, Facial Nerve Grading System 2.0; NLF, nasolabial fold; SFGS, Sunnybrook Facial Grading System*.

On the individual level, 46 patients (85.2%) showed an improvement between T1 and T2 due to the SFGS results. Six patients (11.1%) showed no change, and two patients (3.7%) showed a deterioration. Both, the FNGS 2.0 and the SFGS showed the strongest improvement in the nasolabial fold/zygomatic and the oral region. No further change of facial grading was seen in the follow-up between T2 and T3, neither a further improvement nor a deterioration.

### Correlation Analyses

A correlation analysis was performed for the changes between T1 and T2 for the total/composite scores in relation to the SFGS. The FNGS 2.0 showed the highest correlation (*r* = 0.812, *p* ≤ 0.001), followed by the House-Brackmann grading (*r* = 0.511; *p* ≤ 0.001). The correlation of the SFGS to the Stennert index was the lowest (*r* = 0.318; *p* = 0.09). Neither the age of the patient (*r* = 0.168; *p* = 0.224), the gender (*r* = 0.126; *p* = 0.363) nor the length of the interval between onset of the palsy and training start (*r* = 0.011; *p* = 0.886) correlated the changes of the SFGS between T1 and T2.

## Discussion

Synkinesis typically becomes clearly apparent about 6 months after pathological reinnervation and the full picture is reached after about 12 months (1, 27). Although the median time to onset in the present sample was already 31.1 months, 85% of the patients profited from the training. Furthermore, the waiting time analysis clearly showed that synkinesis remained unchanged before training. The follow-up showed that the effects of the training remained stable for at least 6 months. Of course, a randomized trial is needed to confirm the effectiveness of this short but very intensive training. The presented combined EMG and visual biofeedback training differs significantly in the number of hours per day from other biofeedback-based therapy concepts for patients. Other concepts are typically less intense but are performed over several weeks and months (14, 28–30). The structure of the presented intensified combined electromyography and visual feedback training was based on the established training concept of a constraint-induced movement rehabilitation program for patients after stroke. In accordance to the present results, it has already been shown that compact intensive training over a period of 2 weeks results in an improvement in function in stroke patients with paretic body parts mismatch (16–18). So far, no comparative studies on optimal training frequency and duration of an EMG-feedback approach for patients with synkinesis have been performed (5). This should be the subject of future research.

There are only a few other studies which included an EMG biofeedback element and investigated patients with synkinesis. The treatment time was always much longer than in the present study. The effectiveness of a complex EMG biofeedback and mirror feedback training in comparison to mirror feedback training alone was examined by Ross et al. (11). Training took place every 1–2 weeks for a total of 1 year. Both groups showed statistically significant improvements in facial motility compared to a control group but outcome in-between both therapy groups was not different. Cronin et al. examined an EMG biofeedback training that took place every 1–2 weeks for several months. Their therapy was associated with significant functional improvements of the face, including an increase in symmetry and motility, as well as a reduction in synkinesis (13). Both studies did not include follow-up examination after end of therapy. Long-term effects were only studied after mime therapy. Mime therapy includes massages, relaxation exercises, inhibition of synkinesis, and emotional expression exercises but not EMG-feedback elements (31). The mime therapy effects remained stable even 1 year after therapy. According to Fitts and Posner (32), the learning of motor skills is divided into three stages. At the cognitive level, the trainee, here the patients with synkinesis, must first understand the type of task and learn to perform it (e.g., in the present training: lifting of the affected corner of the mouth). Therefore, the visual feedback and the control by the therapist is important. In the subsequent association stage, this ability must be refined by repeating it and combining it with other abilities (e.g., smiling, i.e., lifting of the affected corner of the mouth together with the contralateral corner). Here, especially the EMG feedback is very important. The level of autonomy is reached when the patient is finally able to integrate the learned ability freely into complex actions (e.g., speaking and smiling with integrated abduction of the corner of the mouth) in activities of daily live. Finally, the intensity of the training with repetitions over hours is very important. The ability or the training effect remains stable if this form of autonomous use is continued permanently (33, 34). Patients with facial palsy are typically extremely motivated to improve their restricted facial motor skills and to integrate what they have learned into their everyday lives (31).

Beyond physical therapy, botulinum toxin is a mainstay of synkinesis therapy (5, 35). Botulinum toxin treatment can also be combined with biofeedback rehabilitation (36). It might be an option to combine our treatment approach with botulinum toxin injection to facilitate specific movement tasks, but this has not yet been evaluated.

Furthermore, we need to introduce objective measurement tools to evaluate the outcome. The SFGS is a very robust but subjective facial grading tool (37). Like in many other studies, the scales were evaluated in the present study only by one examiner. First automated tools feasible for the use in clinical routine are published (22, 38). Such tools should be applied for SFGS or any other grading in future studies. As the present therapy is dependent on the surveillance of a trained therapy, it will also be of interest to develop a remote rehabilitation concept. Therefore, we will need remote EMG devices and especially a simplification of the camera technology using conventional smart phones or, for instance, special remote activity eye wear (39, 40).

## Conclusion

This longitudinal study on 54 patients with post-paralytic facial synkinesis over four points in time showed that facial nerve function did not change during the waiting time before start of the training. An intensified daily training over 10 days using a combined EMG and visual biofeedback setting improved facial grading especially by reducing synkinesis. Finally, the effects remained stable over 6 months. Future studies should validate the results in an external cohort of patients and compare the presented treatment to other approaches at best in randomized controlled trial. Furthermore, it has to show that the treatment is also effective from the patients' perspective, i.e., by the use of patient-related outcome measures using facial paralysis related quality of life assessment tools.

## Data Availability Statement

The raw data supporting the conclusions of this article will be made available by the authors, without undue reservation.

## Ethics Statement

The studies involving human participants were reviewed and approved by Ethics Committee of the Jena University Hospital. The patients/participants provided their written informed consent to participate in this study. Written informed consent was obtained from the individual(s) for the publication of any potentially identifiable images or data included in this article.

## Author Contributions

GV, OG-L, and CD: design of the work. BR, A-MK: data acquisition. GV, OG-L, KG, and CK: analysis. GV, BR, OG-L, CD, and KG: interpretation. All authors draft contribution, approval of the final version to be published, agreement to be accountable for all aspects of the work in ensuring that questions related to the accuracy or integrity of any part of the work are appropriately investigated and resolved.

## Funding

OG-L acknowledges support by a Deutsche Forschungsgemeinschaft (DFG) grant GU-463/12-1.

## Conflict of Interest

The authors declare that the research was conducted in the absence of any commercial or financial relationships that could be construed as a potential conflict of interest.

## Publisher's Note

All claims expressed in this article are solely those of the authors and do not necessarily represent those of their affiliated organizations, or those of the publisher, the editors and the reviewers. Any product that may be evaluated in this article, or claim that may be made by its manufacturer, is not guaranteed or endorsed by the publisher.
