# Supplementary material for: Effect of an Intensified Combined Electromyography and Visual Feedback Training on Facial Grading in Patients With Post-paralytic Facial Synkinesis
Source: Front Rehabil Sci. 2021 Oct 14;2:746188. doi: 10.3389/fresc.2021.746188 (PMC9397686; doi:10.3389/fresc.2021.746188)
Supplement: Supplementary file 1 [file Data_Sheet_1.pdf]

Supplementary Table 1: More details of the combined electromyography and visual feedback training

Effect of an intensified combined electromyography and visual feedback training on facial grading in patients with postparalytic facial synkinesis

G.F. Volk[1,2], B. Roediger[1], K. Geißler[1,2], A.-M. Kuttenreich[1,2], C.M. Klingner[2,3], C. Dobel[1,2], O. Guntinas-Lichius[1,2]

[1]Department of Otorhinolaryngology, Jena University Hospital, Jena, Germany

[2]Facial Nerve Center, Jena University Hospital, Jena, Germany

[3]Department of Neurology, Jena University Hospital, Jena, Germany

Correspondence to: Orlando Guntinas-Lichius

Department of Otorhinolaryngology

Jena University Hospital

Am Klinikum 1

D-07747 Jena, Germany

Tel. +49-3641-9329301

Fax. +49-3641-9329302

Email: orlando.guntinas@med.uni-jena.de

**Supplementary Table 1**. Combined electromyography and visual feedback training*		
**Parameter**	**Target / Classification**	**Description**
Target 1	Training of the activation of complete or partly paralyzed muscles of the affected hemiface	The patient is asked to slightly tense the corresponding muscle and hold this state for a few seconds. Then the muscle in question is relaxed as much as possible. In order to report back even the smallest activities of the paralyzed facial muscle and thus positively strengthen the patient's training efforts, the EMG feedback threshold in the visual or acoustic representation is kept low.<br><br>The therapist pursues the goal of strengthening the muscles of the affected side through intensive training, while the muscles of the healthy half of the face remain largely inactive.
Target 2	Reduction of over-activation of a muscle on paralyzed or contralateral side.	The goal is to get a weaker EMG feedback signal while this muscle is active.<br><br>The therapist pursues the goal of weaken the muscle activation of the affected or contralateral side through intensive training, while the muscles of the other side remain largely inactive
Target 3	Reduction of synkinetic activity	The patients learn to influence and balance the activity of the synkinetically coupled muscles. This is achieved by having the patient contract one muscle while relaxing the other at the same time. Alternatively, the patient has to contract or relax one muscle while the other muscle maintains activity.<br><br>The therapist pursues the goal of weaken the unintended muscle co-activation on the affected side through intensive training, while the muscle with intend use is activated.

List of movements; Selection of exercises related to the therapy aims and problems of the patient	Closing the eyes<br>Closed smile<br>Open smile<br>Kissing mouth<br>Showing teeth<br>Forming of an “O” and “U”<br>Letter exercise "A, E, I, O, U"<br>Move the angle of the mouth individually outwards, backwards and upwards<br>Bloating checks	Sequence, not feasible for all movements : normal side, affected side, bilateral<br>The patient is asked to slightly tense the corresponding muscle and hold this state for a few seconds. Then the muscle in question is relaxed as much as possible. The task is repeated.
List of target muscles for EMG recording, individually selected related to target and task.	M. risorius<br>M. orbicularis oris<br>M. buccinators<br>M. zygomaticus major<br>M. zygomaticus minor<br>M. levator anguli oris<br>M. levator labii superioris alaeque nasi<br>M. mentalis	EMG feedback from two selected muscles.<br>Depending on the task: both unilateral or unilateral and contralateral

*References: See main article.
