# Supplementary material for: Effect of an Intensified Combined Electromyography and Visual Feedback Training on Facial Grading in Patients With Post-paralytic Facial Synkinesis
Source: Front Rehabil Sci. 2021 Oct 14;2:746188. doi: 10.3389/fresc.2021.746188 (PMC9397686; doi:10.3389/fresc.2021.746188)
Supplement: Supplementary file 2 [file Data_Sheet_2.pdf]

Supplementary Table 2: Overview about the four facial grading systems

Effect of an intensified combined electromyography and visual feedback training on facial grading in patients with postparalytic facial synkinesis

G.F. Volk[1,2], B. Roediger[1], K. Geißler[1,2], A.-M. Kuttenreich[1,2], C.M. Klingner[2,3], C. Dobel[1,2], O. Guntinas-Lichius[1,2]

[1]Department of Otorhinolaryngology, Jena University Hospital, Jena, Germany

[2]Facial Nerve Center, Jena University Hospital, Jena, Germany

[3]Department of Neurology, Jena University Hospital, Jena, Germany

Correspondence to: Orlando Guntinas-Lichius

Department of Otorhinolaryngology

Jena University Hospital

Am Klinikum 1

D-07747 Jena, Germany

Tel. +49-3641-9329301

Fax. +49-3641-9329302

Email: orlando.guntinas@med.uni-jena.de

Supplementary Table 2. Overview about the four facial grading systems*

Grading system	Categories		Comment
House-Brackmann (HB) facial nerve grading system	I - Normal<br>II - Mild dysfunction (slight weakness, normal symmetry at rest)<br>III - Moderate dysfunction (obvious but not disfiguring weakness with synkinesis, normal symmetry at rest) Complete eye closure w/ maximal effort, good forehead movement<br>IV - Moderately severe dysfunction (obvious and disfiguring asymmetry, significant synkinesis) Incomplete eye closure, moderate forehead movement<br>V - Severe dysfunction (barely perceptible motion)<br>VI -Total paralysis (no movement)		Gross system, no category for synkinesis
Stennert Index (SI)	Resting score		One point per item, i.e. maximal ten points, higher score = more dysfunction;<br><br>Synkinesis is not addressed in the Stennert Index. Stennert developed an extra score for synkinesis that is seldom used.
	Bilateral palpebral fissure difference	≥3mm	
	Lower eyelid ectropion	Yes	
	Nasolabial fold loss	Yes	
	Ptosis of labial angle	≥3mm	
	Movement score		
	Palpebral fissure could not close		
	While sleeping	Yes	
	Maximum activation	Yes	

	Showing teeth<br>Upper and lower canines Not visible<br>Upper lateral incisors Not visible<br>Whistle<br>distance between philtrum and corner of the mouth on diseased side compared to healthy side >50%	

<table>
<tr>
<td>Facial Nerve Grading System 2.0 (FNGS 2.0)</td>
<td>
<table>
<tr><th rowspan="2">Score</th><th colspan="4">Region</th></tr>
<tr><th>Brow</th><th>Eye</th><th>NLF</th><th>Oral</th></tr>
<tr><td>1</td><td>Normal</td><td>Normal</td><td>Normal</td><td>Normal</td></tr>
<tr><td>2</td><td>Slight weakness
>75% of normal</td><td>Slight weakness
>75% of normal
Complete closure with mild effort</td><td>Slight weakness
>75% of normal</td><td>Slight weakness
>75% of normal</td></tr>
<tr><td>3</td><td>Obvious weakness
>50% of normal
Resting symmetry</td><td>Obvious weakness
>50% of normal
Complete closure with maximal effort</td><td>Obvious weakness
>50% of normal
Resting symmetry</td><td>Obvious weakness
>50% of normal
Resting symmetry</td></tr>
<tr><td>4</td><td>Asymmetry at rest
<50% of normal
Cannot close completely</td><td>Asymmetry at rest
<50% of normal</td><td>Asymmetry at rest
<50% of normal</td><td>Asymmetry at rest
<50% of normal</td></tr>
<tr><td>5</td><td>Trace movement</td><td>Trace movement</td><td>Trace movement</td><td>Trace movement</td></tr>
<tr><td>6</td><td>No movement</td><td>No movement</td><td>No movement</td><td>No movement</td></tr>
</table>
Secondary movement (global assessment)
<table>
<tr><th>Score</th><th>Degree of movement</th></tr>
<tr><td>0</td><td>None</td></tr>
<tr><td>1</td><td>Slight synkinesis; minimal contracture</td></tr>
<tr><td>2</td><td>Obvious synkinesis; mild to moderate contracture</td></tr>
<tr><td>3</td><td>Disfiguring synkinesis; severe contracture</td></tr>
</table>
Reporting: sum scores for each region and secondary movement
<table>
<tr><th>Grade</th><th>Total score</th></tr>
<tr><td>I</td><td>4</td></tr>
<tr><td>II</td><td>5-9</td></tr>
<tr><td>III</td><td>10-14</td></tr>
<tr><td>IV</td><td>15-19</td></tr>
<tr><td>V</td><td>20-23</td></tr>
<tr><td>VI</td><td>24</td></tr>
</table>
NLF, nasolabial fold.
</td>
<td>FNGS 2.0 includes an subcategory on “secondary movements”, classifying the severity of synkinetic activity; higher synkinesis score = more synkinetic dysfunction;

Higher total score = higher facial dysfunction</td>
</tr>
</table>

Sunnybrook Facial Grading System (SFGS)	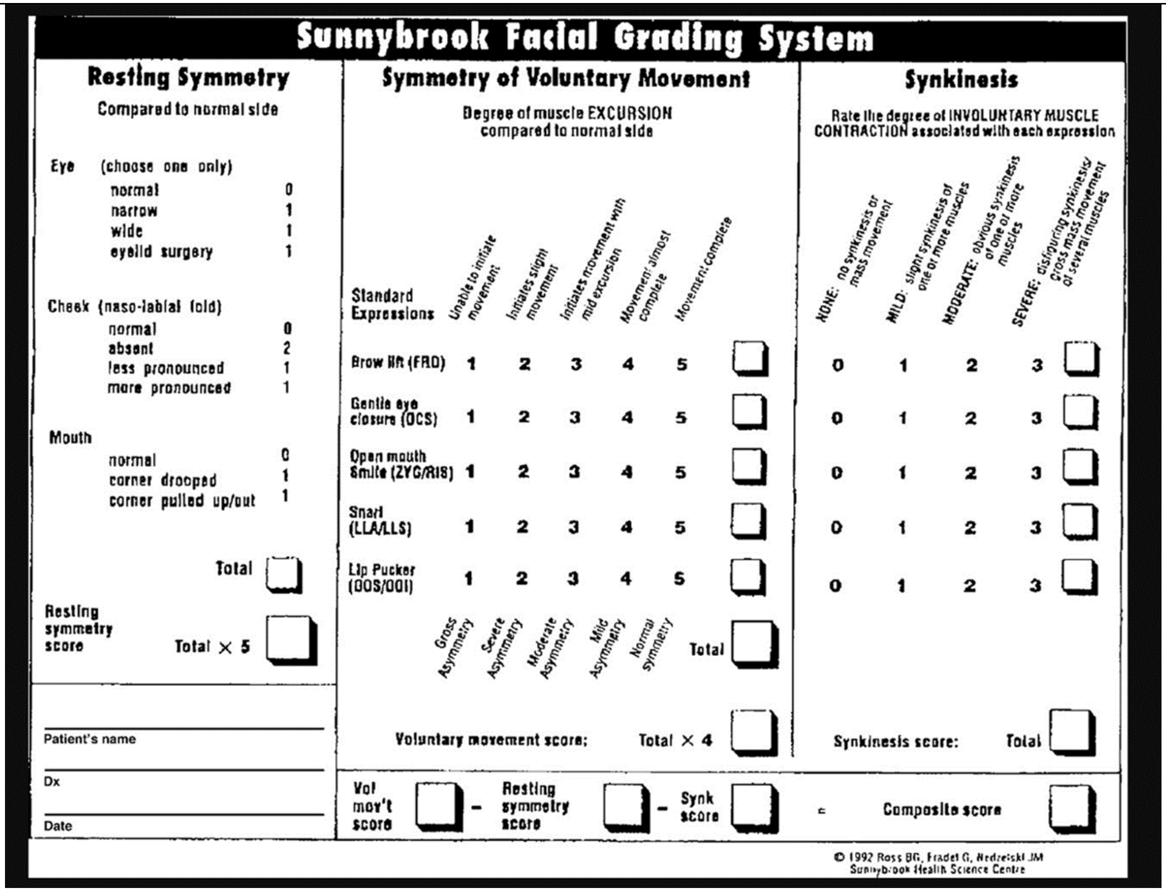	SFGS 2.0 includes an subcategory on“synkinesis, classifying the severity of synkinetic activity; higher synkinesis score = more synkinetic dysfunction;<br><br>BUT: Higher total score = <u>less</u> facial dysfunction

Sunnybrook Facial Grading System

Resting Symmetry

Compared to normal side

Eye (choose one only)	
normal	0
narrow	1
wide	1
eyelid surgery	1

Cheek (naso-labial fold)	
normal	0
absent	2
less pronounced	1
more pronounced	1

Mouth	
normal	0
corner drooped	1
corner pulled up/out	1

Total ☐

Resting symmetry score Total × 5 ☐

Patient's name

Dx

Date

Symmetry of Voluntary Movement

Degree of muscle EXCURSION compared to normal side

Standard Expressions	Unable to initiate movement	Initiates slight movement	Initiates movement with mild excursion	Movement almost complete	Movement complete	
Brow lift (FRO)	1	2	3	4	5	☐
Gentle eye closure (OCS)	1	2	3	4	5	☐
Open mouth Smile (ZYG/RIS)	1	2	3	4	5	☐
Snarl (LLA/LLS)	1	2	3	4	5	☐
Lip Pucker (OOS/OOI)	1	2	3	4	5	☐
	Gross Asymmetry	Severe Asymmetry	Moderate Asymmetry	Mild Asymmetry	Normal symmetry	Total ☐

Voluntary movement score: Total × 4 ☐

Synkinesis

Rate the degree of INVOLUNTARY MUSCLE CONTRACTION associated with each expression

NONE: no synkinesis or mass movement	MILD: slight synkinesis of one or more muscles	MODERATE: obvious synkinesis of one or more muscles	SEVERE: disfiguring synkinesis/ gross mass movement of several muscles	
0	1	2	3	☐
0	1	2	3	☐
0	1	2	3	☐
0	1	2	3	☐
0	1	2	3	☐

Synkinesis score: Total ☐

Vol mov't score ☐ – Resting symmetry score ☐ – Synk score ☐ = Composite score ☐

© 1992 Ross BG, Fradet G, Nedzelski JM
Sunnybrook Health Science Centre

*References: See main article.
